# Biological pretreatment of corn stover for enhancing enzymatic hydrolysis using *Bacillus* sp. P3

**DOI:** 10.1186/s40643-021-00445-8

**Published:** 2021-09-27

**Authors:** Yanwen Wu, Haipeng Guo, Md. Shafiqur Rahman, Xuantong Chen, Jinchi Zhang, Yun Liu, Wensheng Qin

**Affiliations:** 1 Co-Innovation Center for Sustainable Forestry in Southern China, Jiangsu Province Key Laboratory of Soil and Water Conservation and Ecological Restoration, Nanjing Forestry University, Nanjing, 210037 China; 2 Department of Biology, Lakehead University, Thunder Bay, ON P7B 5E1 Canada; 3 School of Marine Sciences, Ningbo University, Ningbo, 315211 China; 4 Department of Microbiology, University of Chittagong, Chittagong, Bangladesh; 5 Beijing Key Laboratory of Bioprocess, College of Life Science and Technology, Beijing University of Chemical Technology, Beijing, 100029 China; 6 Present Address: Learning Support Team, St Margaret’s School, Victoria, BC V8X 3P7 Canada

**Keywords:** *Bacillus* sp. P3, Biological pretreatment, Corn stover, Enzymatic hydrolysis, Thermoalkalotolerant enzymes

## Abstract

The biological pretreatment for the enzymatic hydrolysis of lignocellulosic biomasses depends exclusively on the effective pretreatment process. Herein, we report a significant enhancement of enzymatic saccharification obtained with corn stover using a bacterial strain *Bacillus* sp. P3. The hemicellulose removal from corn stover by the strain *Bacillus* sp. P3 was evaluated for enhancing subsequent enzymatic hydrolysis. Therefore, our study revealed that an alkaline-resistant xylanase as well as other enzymes produced by *Bacillus* sp. P3 in fermentation broth led to a substantially enhanced hemicellulose removal rate from corn stover within pH 9.36–9.68. However, after a 20-day pretreatment of corn stover by the strain P3, the glucan content was increased by 51% and the xylan content was decreased by 35%. After 72 h of saccharification using 20 U/g of commercial cellulase, the yield of reducing sugar released from 20-day pretreated corn stover was increased by 56% in comparison to the untreated corn stover. Therefore, the use of the strain P3 could be a promising approach to pretreat corn stover for enhancing the enzymatic hydrolysis process of industrial bioenergy productions.

**Supplementary Information:**

The online version contains supplementary material available at 10.1186/s40643-021-00445-8.

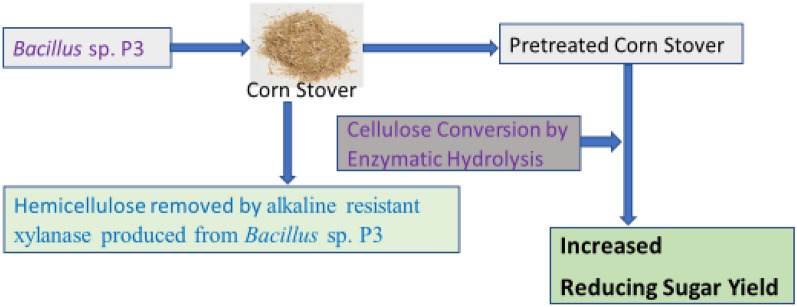

## Introduction

Increasing consumption of fossil fuel has resulted in an increase in fuel cost and atmospheric greenhouse gas emission, thus the raw materials for fuel production are gradually being replaced by renewable bioresources. Lignocellulosic biomasses from agricultural residues are a great potential resource for biofuel production due to their wide distribution, abundant reserve, low price, and renewability (Wan and Li 2010). Therefore, the utilization of these lignocellulosic biomasses to produce biofuels has become an interesting research field. However, various obstacles associated with the current methods of biofuel production using lignocellulosic biomasses still need to be overcome. One of the key problems hampering the bioconversion of agricultural residues is the high resistance of lignocellulose to hydrolysis, which is caused by the recalcitrant crystalline structure of cellulose fibrils surrounded by hemicelluloses and further sealed by lignin (Himmel et al. 2007), thus effective pretreatment processes are necessary to enhance the hydrolysis rate and separate hemicellulose from cellulose that allow the access of hydrolytic enzymes and increase the sugar yield (Zhao et al. 2012; Kumar et al. 2009).

Therefore, chemical pretreatment methods include steam explosion, acid hydrolysis, alkaline wet oxidation, and ammonia fiber expansion (AFEX) for efficient degradation of hemicellulose require expensive instruments and high energy consumption (Wang et al. 2013). Furthermore, these chemical pretreatments often result in the generation of inhibitors as well as the production of acidic or alkaline wastewater, which create an environmental issue. Therefore, an eco-friendly biological pretreatment using microorganisms has been considered as an alternative to harsh chemicals and thermochemical pretreatments for lignocellulosic biomass conversion due to its low energy requirement (Keller et al. 2003). The microbial pretreatment involving fungi, especially white, brown, and soft-rot fungi have been widely used to degrade hemicellulose and lignin from lignocellulosic biomasses (Keller et al. 2003). The white-rot fungi have been studied as the most effective microbial candidates to pretreat lignocellulosic biomasses (Kirk and Cullen 1998). The reducing sugars released from pretreated lignocellulosic biomasses (wheat straw, rice straw and corn stover) increased 50–65% enzymatic saccharification compared to that of non-pretreated control samples using white-rot fungi (Wang et al. 2013; Bak et al. 2010). Although a wide variety of hydrolytic enzymes (cellulases and hemicellulases) from fungal strains have been employed in the industry (Kirk and Cullen 1998; Pérez et al. 2002), the bacterial strain could be used as a more promising candidate to pretreat the lignocellulosic biomasses due to their fast growth and cellulolytic enzyme-producing ability in harsh environmental conditions which are advantageous in lignocellulose degradation (Lynd et al. 2002). Moreover, compared to fungi, bacteria have the following advantages (Sangrila and Maiti 2013): (1) bacteria have a higher growth rate to accelerate the production of enzymes; (2) bacteria can produce more complex glycoside hydrolases which have the potential to provide synergistic functions; (3) bacterial strains are excellent at resisting environmental stresses.

In pulp, paper, leather and textile industries, cellulase enzymes need to perform under harsh conditions including high temperature, alkaline and detergents ambiance which can cause denaturing of proteins and loss of catalytic activity (Zamost et al. 1991; Sondhi et al. 2015). Therefore, in harsh conditions, most of the existing enzymes perform very poorly. Moreover, lignocellulolytic enzymes which are resistant to alkaline pH are important and demanding in today’s Kraft-pulp industry. Thus, the isolation of alkali-tolerant lignocellulolytic enzymes producing microbes is necessary and essentially useful to produce lignocellulolytic enzymes. The optimal pH values of the fungal culture for maximum cellulase production are in slightly acidic ranges from 4.8 to 6.0 (Chang and Steinkraus 1982). However, the optimal pH values of the most cellulolytic enzyme-producing bacteria are in a range from 8.0 to 9.5 (Liu et al. 2017), thus alkali-tolerant bacterial candidates are needed for enzymatic hydrolysis of cellulose. Moreover, at an unsuitable pH condition, the biodegradation efficiency of lignocellulosic biomass will be decreased due to low enzyme activities of the microorganism (Maki et al. 2009). This problem severely hinders the direct application of cellulolytic enzymes producing bacteria to pretreat the lignocellulosic biomasses for biofuels production. Consequently, to improve the biomass degradation efficiency, it is necessary to find some alkali-tolerant cellulolytic enzymes from bacterial candidates.

Therefore, our study was focused on an alkali-tolerant cellulolytic enzyme-producing *Bacillus* sp. P3 strain to explore the feasibility of pretreatment for the degradation ability and enzymatic productivity using various lignocellulosic materials. The lignocellulosic material that induced maximum enzyme production was selected as the carbon source to optimize the enzyme production conditions. Under optimal conditions, the strain P3 was directly cultured with the selected lignocellulosic material to weaken its structure mainly by decomposing hemicellulose. The effect of pretreatment was then assessed by reducing sugar yields from the pretreated materials in enzymatic saccharification with commercial cellulase.

## Materials and methods

### Corn stover and bacterial strain P3

Corn stover was provided by Agriculture and Agri-Food Canada. The air-dried corn stover was chopped and milled to pass through a 50-mesh sieve for this study. The milled sample was stored at ambient temperature in an airtight container until use.

The *Bacillus* sp. strain P3 (Accession No. MF462257) and its enzymatic characteristics have been described previously (Guo et al. 2017a). The strain P3 was stored at − 70 °C. Moreover, one set of this strain P3 was maintained at 4 °C on Luria-Bertani (LB) agar (10 g/L peptone, 5 g/L yeast extract, 10 g/L NaCl and agar 15 g/L) slant and sub-cultured every 2 weeks. Seed culture for batch fermentation experiments was prepared from the stock slant culture by inoculating into LB broth (10 g/L peptone, 5 g/L yeast extract, 10 g/L NaCl) medium, incubated at 37 °C and 200 rpm for 12 h.

### Evaluations of biomass degradation abilities

To assess the biomass degradation ability of the strain P3, different lignocellulosic biomasses including agave, algae, corn stover, *Miscanthus*, wheat bran, wood dust and pine chip were used as the carbon sources according to the method described by Guo et al. (2017b). Briefly, a 5.0 μL of the overnight-grown LB broth culture was dropped or inoculated on the plate containing modified minimal salt (MMS) medium (0.1% NaNO_3_, 0.1% K_2_HPO_4_, 0.1% KCl, 0.05% MgSO_4_, 0.05% yeast and 0.3% peptone) supplemented with 1.5% agar and 0.5% biomass or CMC or xylan, incubated at 37 °C for 48 h. The biomass degradation ability was evaluated based on the size of biomass hydrolyzing zone (zone of clearance or halo zone) produced on the plate by the bacterial strain after staining with Gram’s iodine solution.

### Optimization of fermentation parameters for cellulolytic enzyme production

To optimize the fermentation conditions for maximum enzyme production, the strain P3 was inoculated (2%, v/v) in the MMS broth medium supplemented with 0.5% (w/v) biomass or CMC, incubated at 37 °C for 12, 24, 36, 48, 60 and 72 h, respectively, in a rotary shaker incubator of 200 rpm. To evaluate the influence of different carbon sources on enzyme production, the agave, algae, corn stover, *Miscanthus*, wheat bran, wood dust, pine chip and CMC were used as the substrates according to the method described previously (Guo et al. 2017b). Following incubation, the broth culture was centrifuged at 12,000*g* for 3 min to obtain the supernatant, which was used as the crude enzymes for CMCase and xylanase activities analysis. The activities of CMCase and xylanase were determined by measuring the released reducing sugar from substrate. The reducing sugar content was measured by DNS method (Miller 1959). The substrate corn stover (carbon source) was selected to optimize the biomass concentrations for lignocellulosic enzymes production due to its high CMCase and xylanase production ability compared with other biomasses tested herein. For optimization of corn stover concentrations, the MMS broth medium was supplemented with 0.5%, 1.0%, 2.0% and 4.0% corn stover, respectively.

The influences of temperature on CMCase and xylanase productions were investigated by culturing the strain P3 at 30, 37, 45 and 50 °C for 24 h, while the effects of different initial pH values of the culture medium on enzyme productions were determined in a wide range of pH from 5.0 to 10.0 at an interval of 0.5. The pH values of medium were adjusted with the addition of hydrochloric acid (HCl) and sodium hydroxide (NaOH).

### Effects of temperature and pH on enzyme activities

The crude enzymes harvested under optimal fermentation conditions were taken for evaluating the effects of temperature and pH on CMCase and xylanase activities. However, for determining the effects of incubation temperature and pH of the reaction mixtures with crude enzyme, a wide range of temperatures from 40 to 80 °C and pH from 4.0 to 9.5 were set the incubation periods. The 0.05 M citrate and Tris–HCl buffer solutions were used, respectively, to set the pH of the enzyme reaction mixtures.

### Bacterial pretreatment of corn stover

For bacterial pretreatment, dried corn stover (0.5%, w/v) was mixed with MMS broth medium, autoclaved at 121 °C for 30 min. An overnight LB broth culture of the strain P3 was inoculated (2%, v/v) in an Erlenmeyer flask containing 50 mL of medium and a control flask of 50 mL medium without bacterial inoculum was also subjected to the same conditions. Bacterial pretreatment was performed at 37 °C with 200 rpm for 20 days, and all flasks were covered by parafilm to prevent water evaporation in this process (Papavizas et al. 1984). The samples were taken at 5- to 10-day intervals, filtered through a double-layered muslin cloth (300 mesh) and the supernatant was collected for the determination of reducing sugar as well as enzymes (CMCase and xylanase) activities. The residue was washed several times with distilled water through a double-layered muslin cloth (300 mesh) to remove the bacterial cells, dried at 50 °C until constant weight and used to determine the weight loss and cell wall compositions. The pH, enzyme activities and reducing sugar content of supernatant were determined after centrifugation at 1200*g* for 3 min.

### Biomass composition analysis

According to the methods described by Shrestha et al. (2015), the analysis of cellulose and hemicellulose contents was carried out by measuring the contents of glucan and xylan. Anthrone–sulfuric acid and orcinol–hydrochloric acid methods (Leyva et al. 2008; Tomoda 1963) were used to determine the content of hexose and pentose, respectively. The Klason lignin analysis was conducted using the method written by Ibáñez and Bauer (2014).

### Enzymatic saccharification of pretreated corn stover

To detect the saccharification effect of the corn stover pretreated by the strain P3, commercial cellulase extracted from *Trichoderma reesei* ATCC 26921 (Celluclast 1.5 L, Novozymes, Franklinton, NC, USA) was used. The corn stover samples pretreated for 5, 10 and 20 days were saccharified by loading 20 FPU/g of commercial cellulase, the amount of which was set at the maximum to sufficiently hydrolyze the substrate according to the previous study (Singh et al. 2009). Enzymatic saccharification was conducted in 0.05 M citrate buffer (pH 4.8) containing 1% (w/v) pretreated corn stover and 0.005% sodium azide (Ferraz et al. 2017). The reaction mixture was incubated at 50 °C with an agitation of 200 rpm for 72 h. The non-pretreated corn stover was used as the control group. The reducing sugar was determined using method DNS method (Miller 1959).

### Statistical analysis

All data in our experiments were obtained from the mean of three replicates. To quantify the significant difference between different treatments, statistical analysis was carried out by one-way analysis of variance. Pearson correlation analysis was conducted to explain the main factor resulting in the release of reducing sugar in enzymatic saccharification. Statistical analysis was performed using SPSS (SPSS Inc., USA, version 13.0).

## Results and discussion

### Biomass degradation ability of *Bacillus* sp. P3

**Fig. 1:**
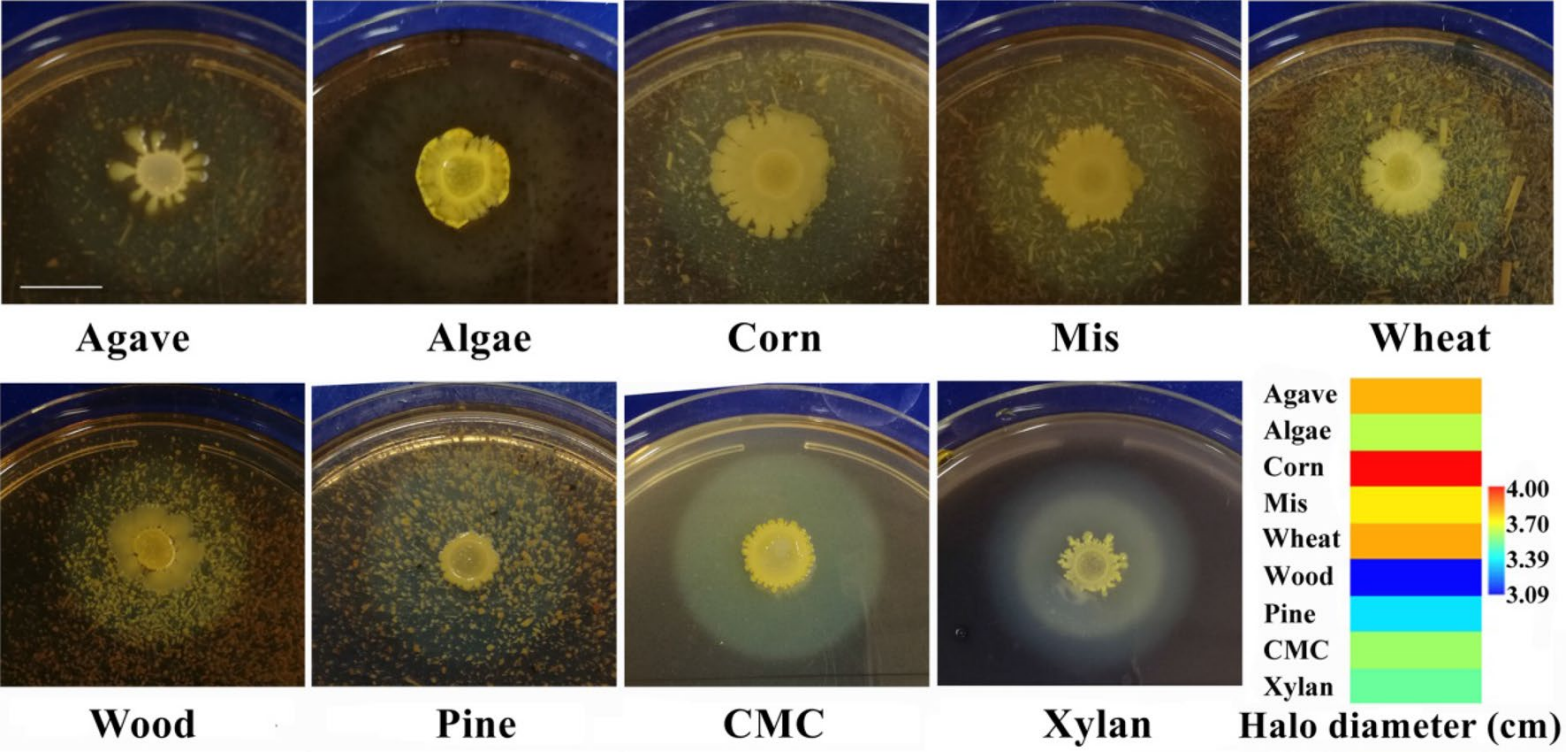
The halo diameters (cm) were produced by of *Bacillus* sp. P3 using on MMS agar medium supplemented with 0.5% different biomasses. The hydrolysis abilities exhibited by the strain P3 were compared by matrix plot

*Bacillus* sp. P3 has the capability of producing cellulolytic enzymes by utilizing various lignocellulosic materials. The biomass hydrolysis ability of the strain P3 was evaluated on the cellulosic biomass or CMC or xylan-containing MMS agar culture plate by staining with Gram’s iodine solution (Fig. 1). Gram’s iodine produces bluish-black products with cellulose but not with its hydrolysates, therefore, a clear zone produced around the bacterial growth after addition of iodine solution indicates that the organism has hydrolyzed cellulose (Guo et al. 2017b). The strain P3 produced the clear or halo zones, ranging from 3.09 to 4.02 cm on the plates by hydrolyzing the biomasses, and the maximum hydrolysis (4.02 cm halo zone) of biomass was detected using corn stover as a carbon source (Fig. 1). Moreover, in our experiment, the clear halo regions on the CMC and xylan-containing plates indicated the hydrolysis of biomasses by CMCase and xylanase enzymes, respectively, produced from the stain P3, and the results are supported by the result reported previously (Lin et al. 2017). Several studies have been done by other researchers on the capability of producing various extracellular lignocellulolytic enzymes by *Bacillus* sp. (Wilson 2009; Arantes and Saddler 2010), and the enzymes exhibited outstanding hydrolytic ability to various lignocellulosic feedstocks (Alvira et al. 2010).

### Optimization of culture conditions for cellulolytic enzymes production

#### Substrates and its concentrations for maximum cellulolytic enzymes production

**Fig. 2:**
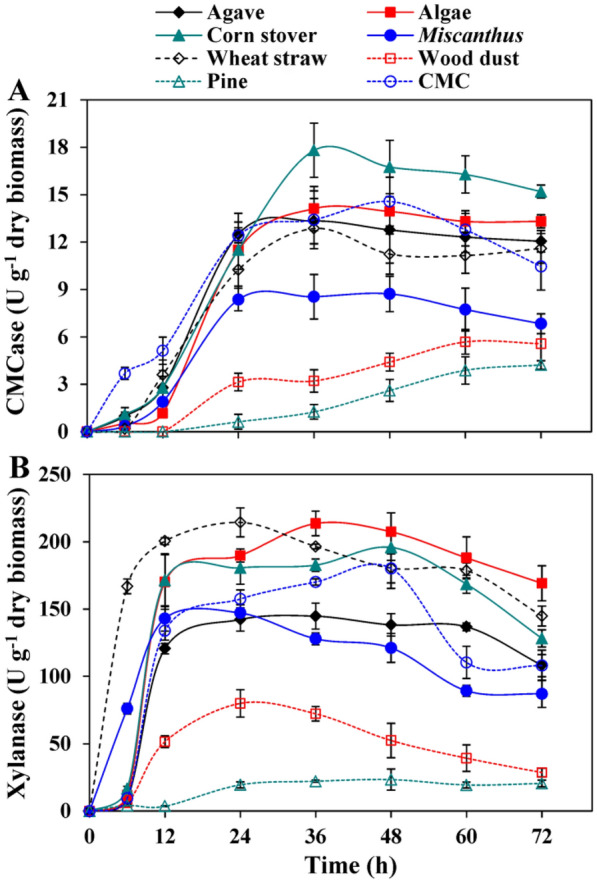
CMCase (**A**) and xylanase (**B**) activities of the *Bacillus* sp. P3 strain cultivated in MMS broth medium at 0.5% (w/v) of different biomasses and CMC. Incubation temperature was 37 °C. Bars indicate the standard deviation (*n* = 3)

Therefore, for selecting the best substrate (carbon source) to induce the production of cellulolytic enzymes, eight typical lignocellulosic biomasses viz., agave, algae, corn stover, *Miscanthus*, wheat bran, wood dust, pine chip and CMC were used for cellulolytic enzyme production by the strain *Bacillus* sp. P3. The maximum CMCase activity with the value of 17.81 U/g was obtained using 0.5% (w/v) corn stover after 36 h of cultivation, while the CMCase activities ranged from 4.23 to 14.57 U/g were obtained using other carbon sources (Fig. 2A). In addition, the maximum xylanase activities were 214.41, 213.59 and 195.85 U/g using wheat bran, algae and corn stover as the carbon sources after 24 h, 36 h and 48 h of fermentation, respectively (Fig. 2B). The CMCase and xylanase produced from *Bacillus* sp. strains can be highly induced by the various lignocellulosic biomasses, the main components of which are cellulose, hemicellulose and lignin (Maki et al. 2009; Sadhu et al. 2013). The maximum CMCase activity of the strain P3 was induced by corn stover, which is similar with the results obtained using *Bacillus subtilis* (Akhtar et al. 2001). Moreover, in this study, the highest xylanase activity (195.85 U/g) exhibited by the strain P3 using corn stover as a carbon source was significantly higher than that of *Bacillus licheniformis* A99 (16.30 U/g) induced by wheat bran under optimal fermentation conditions (Archana and Satyanarayana 1997). Therefore, the untreated corn stover was selected as a potential lignocellulosic biomass to perform pretreatment experiments due to its easy degradation and high cellulolytic enzymes production by P3.

**Fig. 3:**
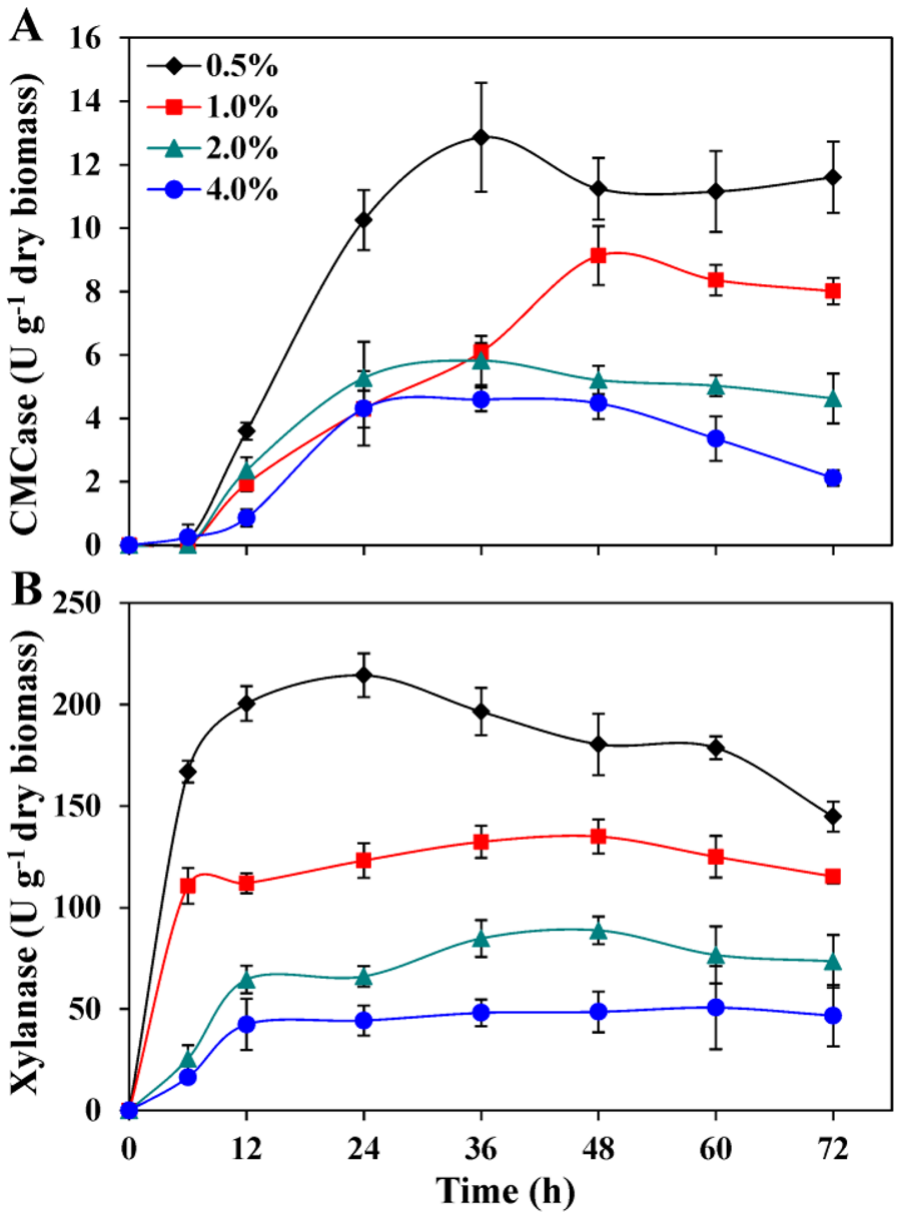
CMCase (**A**) and xylanase (**B**) activities of *Bacillus* sp. P3 in MMS broth medium supplemented with different concentrations of corn stover as a substrate. Incubation temperature was 37 °C. Bars indicate the standard deviation (*n* = 3)

The effects of different concentrations of untreated corn stover on cellulolytic enzymes production were investigated by adding 0.5%, 1.0%, 2.0% or 4.0% (w/v) of corn stover in the MMS broth medium. The maximum CMCase (12.86 U/g) and xylanase (214.41 U/g) produced by the strain P3 using 0.5% corn stover were remarkably higher than the results achieved with other three concentrations after 36 h and 24 h of fermentation, respectively (Fig. 3). The same substrate concentration (0.5%, w/v) to produce cellulolytic enzymes by *Bacillus* sp. strains has been reported earlier (Guo et al. 2017b). Moreover, the activities of CMCase and xylanase were significantly decreased with increasing substrate concentrations. Specifically, the CMCase and xylanase activities obtained with 0.5% corn stover were increased dramatically (Fig. 3). The excessive biomass content effects on oxygen transfer in the fermentation broth, which inhibited the cell growth and enzymatic secretion of bacteria (Guo et al. 2017a). In addition, the high concentrations of several hydrolysates (mannose, xylose, and galactose) produced in the process of cellulosic hydrolysis may also be the reason for restraining the activities of cellulolytic enzymes (Xiao et al. 2004).

#### Optimization of incubation temperature and medium initial pH for maximum enzyme production

To optimize the medium initial pH and fermentation temperature for maximum production of cellulolytic enzymes, the strain P3 was cultured in the MMS broth medium supplemented with 0.5% corn stover for 24 h (Additional file 1: Fig. S1). The optimal temperature of the strain P3 for producing maximum CMCase and xylanase was 37 °C, and the enzyme production was decreased drastically after reached the peak (Additional file 1: Fig. S1A). These results were consistent with an earlier study indicating the optimal temperature of bacterial growth and metabolism was around 37 °C (Huser et al. 1982). Nevertheless, the strain P3 showed the maximum productions of enzymes at an initial pH 7.0; however, more than 60% relative enzyme activities were observed when the medium initial pH ranged from 7.5 to 10.0 (Additional file 1: Fig. S1B). Moreover, the strain P3 exhibited more than 90% relative xylanase activity at the initial pH ranging from 9.0 to 10.0, where the highest relative activity of xylanase was obtained at pH 10.0 (Additional file 1: Fig. S1B). Therefore, our results obtained from *Bacillus* sp. P3 strain were supported by other *Bacillus* sp. strains, which grew and produce xylanases in a wide range of pH (6.0–10.0) (Bansod et al. 1993; Annamalai et al. 2014).

### Optimization of temperature and pH for maximum enzyme activities

**Fig. 4:**
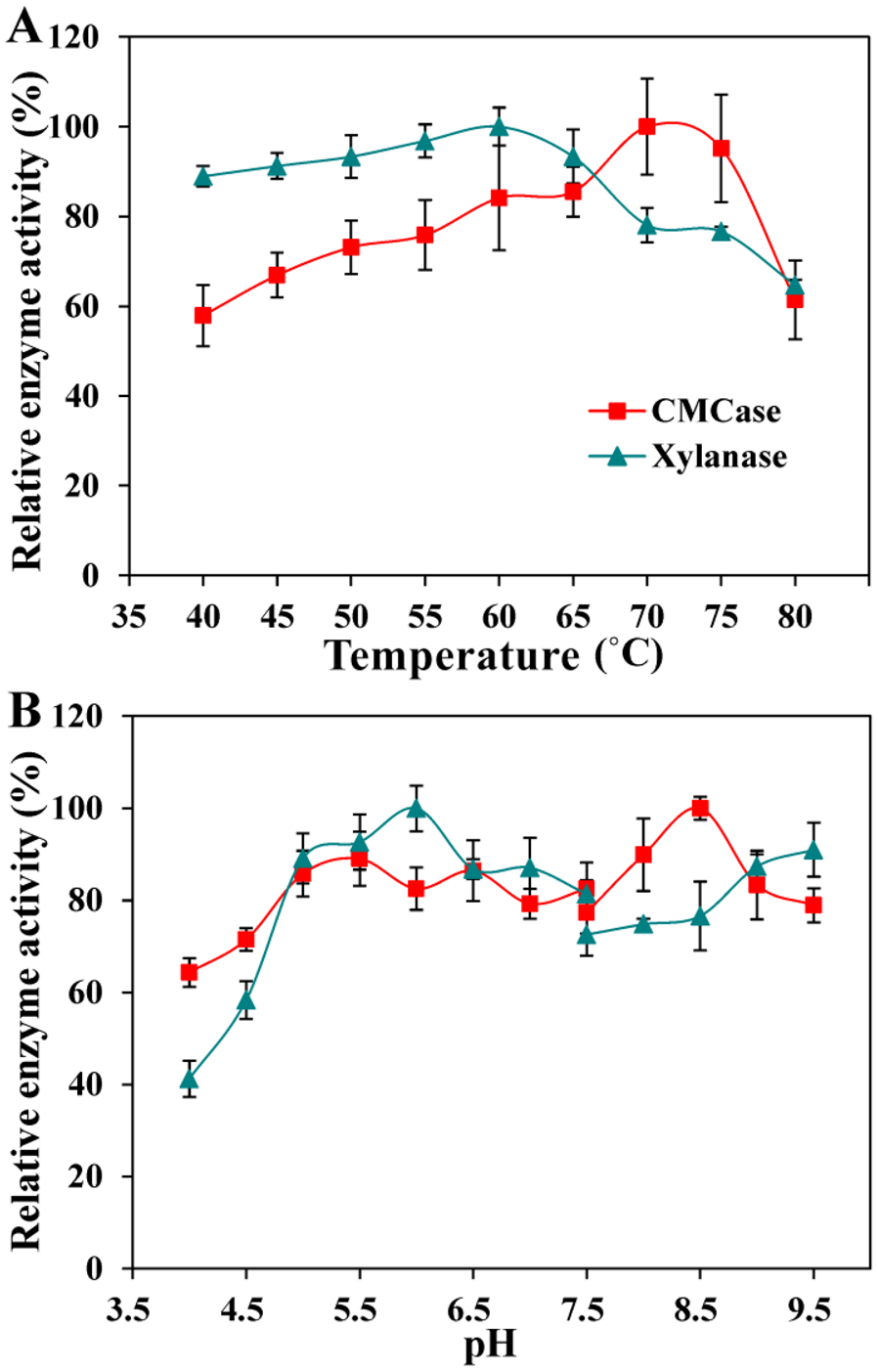
Effects of different temperatures (**A**) and pH (**B**) on enzymatic activities (%) determined by incubating the crude enzymes with CMC or xylan for 30 min. Bars indicate the standard deviation (*n* = 3)

Effects of temperature on the activities of CMCase and xylanase obtained from the strain P3 were assessed at different temperatures ranging between 40 and 80 °C (Fig. 4A). The optimal temperatures for maximum CMCase and xylanase activity were found at 70 and 60 °C, respectively, and both enzymes exhibited more than 60% relative activity with slightly declined after reached the peak value (Fig. 4A). These results presented in Fig. 4A were higher than the optimal temperatures of several thermotolerant cellulolytic enzymes produced by the typical *Bacillus* strains (Kim et al. 2009; Annamalai et al. 2011). Nevertheless, the effects of pH on CMCase and xylanase activities were evaluated using different pH in enzyme reaction mixtures (Fig. 4B). The optimal pH for maximum CMCase activity was recorded as 8.5, and more than 75% relative activity was attained between pH 6.0 and 9.5. Therefore, in case of xylanase, the maximum activity was obtained at a pH 6.0, and more than 70% relative activities were accomplished in alkaline pH conditions (Fig. 4B). The optimal pH values of two cellulolytic enzymes produced by the strain P3 were significantly higher than that of cellulases and xylanases produced by other *Bacillus* sp. strains (Kim et al. 2009; Lee et al. 2008), indicating that cellulolytic enzymes produced by strain P3 were alkalotolerant.

According to an earlier study, lignocellulose deconstructing enzymes (cellulases and hemicellulases) could be used to pretreat lignocellulosic biomass for biofuel production, and the production methods are normally conducted at high temperature (≥ 50 °C) with alkaline conditions to increase the reaction velocities of enzymatic hydrolysis and yields of reducing sugar (Bhalla et al. 2013). Therefore, the application of thermotolerant and alkalotolerant cellulolytic enzymes to high temperature and alkaline environments was put forward as an efficient approach to overcome these limitations (Yeoman et al. 2010). It is widely accepted that bacteria are one of the most efficient producers of thermoalkalotolerant cellulases and xylanases (Saratale and Oh 2011). Several scholars have been tried to isolate potential bacterial candidates for the production of these thermotolerant and alkaliphilic cellulolytic enzymes due to their enormous industrial potential (Maki et al. 2009). Meanwhile, overmuch cellulase would contribute to the loss of cellulose for subsequent saccharification, therefore the strong hydrolytic ability mainly caused by high xylanase activity of a bacterial strain is applicable in biopretreatment for selective removal of hemicellulose from lignocellulosic biomass (Kohli et al. 2001). In this study, the strain *Bacillus* sp. P3 had the capability of withstanding extreme conditions of thermal and high pH and exhibited 16.67-fold higher activity of xylanase compared to that of CMCase in the optimal cultivation condition (Fig. 3). Hence, the strain P3 and its cellulolytic enzymes could be used as the potential candidates for harsh production applications.

### Characterization of fermented supernatants and corn stover before and after pretreatments

#### The pH values, cellulolytic enzyme activities and reducing sugar yields

The pH value of the fermentation broth notably increased from neutral (initial pH 7.0) to high alkaline (9.41) after a 5-day cultivation of the strain P3 and the high pH condition (9.36–9.68) was observed until end of this experiment (Table 1). Most of the *Bacillus* sp. strains have been proven the capability of changing surrounding habitats to be alkaline during the fermentation process due to secretion of secondary metabolites (Schallmey et al. 2004), which could be the main reason to explain the pH variation in this study.

**Table 1:** The pH values, enzyme activities and reducing sugar yields of the supernatants, as well as the weight losses and compositions of corn stover pretreated with strain P3 for different times (*n* = 3)

Time (days)	pH	Enzymes (U/g)		Reducing sugar (mg/g)	Biomass wt. loss (%)	Component (%)		
		CMCase	Xylanase			Glucan	Xylan	Acid-insoluble lignin
0	7.00	0.00	0.00	16.22 ± 1.23	0.00	26.82 ± 1.57	25.98 ± 2.68	21.90 ± 0.20
5	9.41 ± 0.03	13.12 ± 0.82	135.60 ± 2.29	50.23 ± 2.09	30.43 ± 0.33	31.54 ± 1.90	21.74 ± 0.62	22.80 ± 0.40
10	9.68 ± 0.07	14.50 ± 0.55	199.63 ± 7.57	41.17 ± 2.02	32.53 ± 0.38	36.02 ± 0.80	18.51 ± 1.71	21.90 ± 0.42
20	9.36 ± 0.11	12.04 ± 0.76	148.90 ± 5.01	55.50 ± 0.74	33.91 ± 0.21	40.58 ± 2.09	16.90 ± 0.11	18.40 ± 0.28

Under the alkaline condition created by the strain P3, the high activity of xylanase was detected during the process of pretreatment with the maximum values of 199.63 U/g after 10 days of incubation (Table 1). After 20 days of pretreatment, xylanase activity declined with the value of 148.90 U/g. For CMCase activity, a similar trend was observed (Table 1). The highest level of xylanase activity observed here was higher than the activities of enzymes from other biomass-degrading *Bacillus* sp. strains under optimal conditions (Archana and Satyanarayana 1997), thus the strain P3 has an ability for producing higher level of xylanase under alkaline milieu. Moreover, after pretreatment, the reducing sugar released in the culture broth was increased from 16.22 mg/g (0 day) to 55.50 mg/g (20 days) (Table 1). It was obvious that the cellulolytic enzymes produced in alkaline environment (pH 9.36–9.68) by the strain P3 had a significant effect on the release of xylose or glucose from corn stover.

#### The weight losses and compositional variations of corn stover

The weight losses of corn stover decayed by the strain P3 ranged from 30.43 to 33.91% with the increase of pretreatment time (5 to 20 days). Compared to the corn stover pretreated for 5 days, the weight loss increased by only 3.5% after 20 days of pretreatment, which indicated that there was slight consumption of raw materials with the extension of processing time. Moreover, the final weight loss caused by bacterial pretreatment in this study was much lower than the weight losses (38.60–52.80%) caused by efficient biomass-degrading fungi (Saha et al. 2016). Certain microorganisms, which were used to pretreat biomass, could simultaneously degrade lignin and polysaccharides, resulting in unnecessary loss of material. However, the strain P3 is significantly efficient for the pretreatment of corn stover and able to selectively degrade lignin. Thus, the result reported in this study demonstrated that the strain P3 has a great potential to pretreat the corn stover with less material loss.

Therefore, the effects of biopretreatment using the strain P3 on glucan, xylan and lignin contents of the pretreated corn stover were markedly different from non-pretreated corn stover (Table 1). The non-pretreated corn stover contained 26.82% glucan, 25.98% xylan and 21.90% acid-insoluble lignin (Table 1), which were similar with the results obtained from previous research (Liu et al. 2013). After a 5-day pretreatment by the strain P3, the corn stover had a xylan content of 21.74% showing a significant decline and the content of glucan was increased to 31.54% (Table 1), indicating the xylan (hemicellulose) was first decomposed by the high-content xylanase (135.60 U/g) produced by the strain P3, thus the cellulose would then be exposed to CMCase for further utilization (Polizeli et al. 2005). After 20 days of biopretreatment, the glucan content was significantly increased to 40.58%, whereas the concentration of xylan was decreased markedly to 16.90% with slight lignin removal. The ability to produce a large amount of xylanases from xylan present in hemicellulose has been previously observed in several *Bacillus* sp. strains (Sá-Pereira et al. 2002; Subramaniyan and Prema 2000). Therefore, in this study, the exposure of glucan and decomposition of xylan demonstrated a satisfactory effect on the biopretreatment of lignocellulosic biomass (corn stover) for the removal of xylan by the strain P3. Also, the strain P3 exhibited the better xylan removal efficacy from lignocellulosic biomass compared to that of xylanase-producing fungi and physicochemical methods (Kuhar et al. 2008; Ravindran et al. 2017).

### Effects of biological pretreatment on enzymatic saccharification of corn stover

**Fig. 5:**
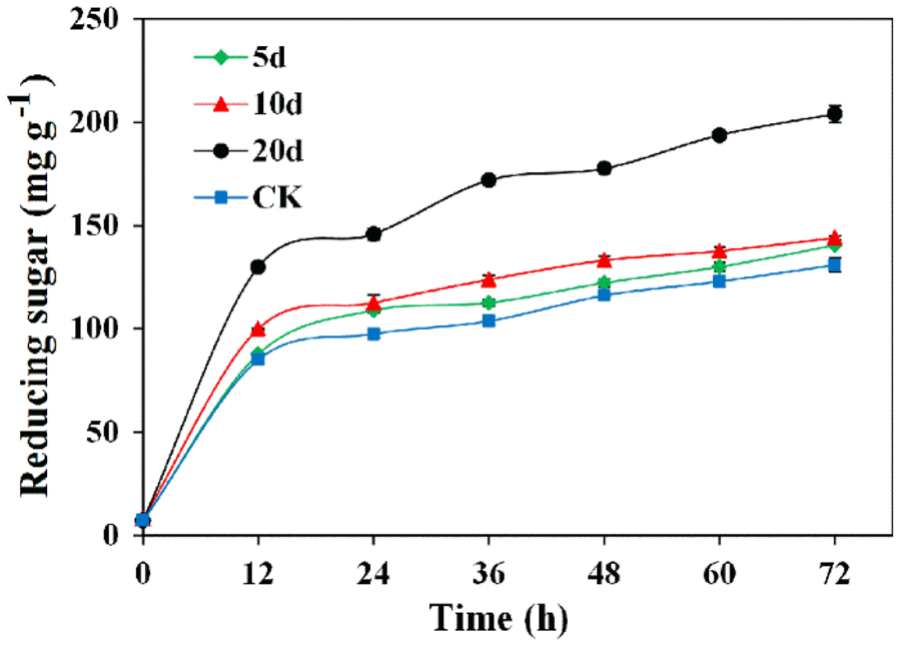
Saccharification of 0, 5, 10 and 20-day pretreated corn stover using commercial cellulases. Bars indicate the standard deviation (*n* = 3)

For efficient hydrolysis of corn stover, commercial cellulase 20 U/g, was used to conduct the enzymatic saccharification. Therefore, 20 U/g cellulase was selected for maximum hydrolyzing of substrate based on the result reported previously (Singh et al. 2009). The bacterial pretreated and non-pretreated corn stover exhibited significant degradability using 20 U/g commercial cellulase (Fig. 5). After 72 h of incubation, the 5, 10 and 20-day pretreated corn stover samples were hydrolyzed by commercial cellulase, yielding 140.51, 144.06 and 203.97 mg/g reducing sugar, respectively, while the corresponding yield of reducing sugar released from non-pretreated corn stover was 130.87 mg/g (Fig. 5). Therefore, in this study, it was clear that the efficiencies of saccharification were significantly increased by 56% after 20 days of pretreatment, which is the highest increase rate of reducing sugar yield. Also, our result was superior to the previous results showed an increase of 32% reducing sugar yield after 60 days of incubation using fungal strains to pretreat lignocellulosic biomass (Taniguchi et al. 2005), thus the strain P3 is a highly efficient bacterial candidate to improve saccharification efficiency of lignocellulosic material. Moreover, according to the results of Pearson correlation analysis (Additional file 1: Fig. S2), the final yields of reducing sugar from pretreated and non-pretreated corn stover had a significant positive correlation with the content of glucan in corn stover (*P* < 0.05). Therefore, it can be inferred that the enzymatic hydrolysis was enhanced greatly after 20 days of pretreatment due to the efficient removal of the hemicellulosic fraction for exposing cellulose polymers to provide more accessible surface areas for commercial cellulase (Wan and Li 2010).

### Overall mass balance

**Fig. 6:**
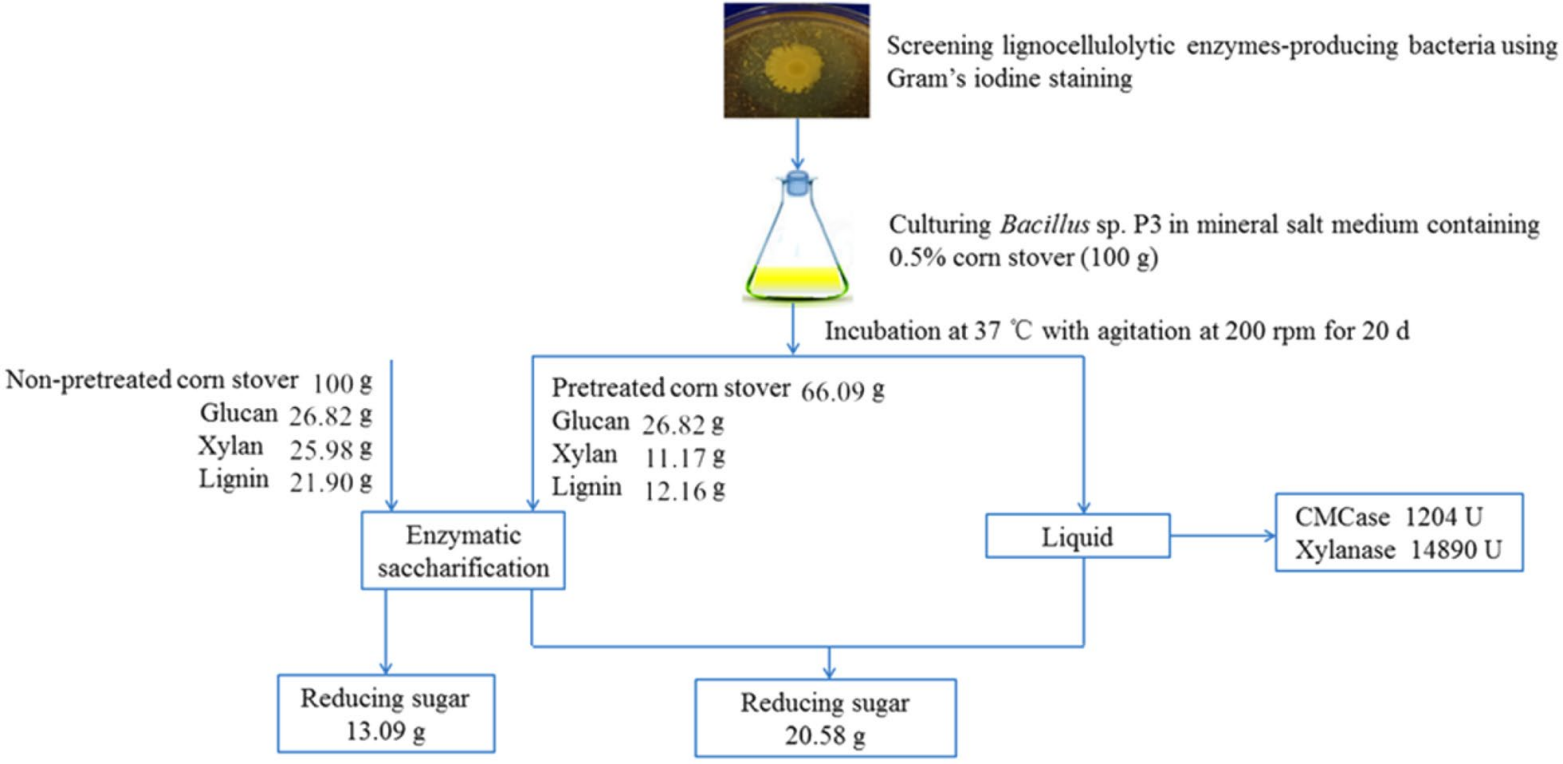
Flowchart summarizing the effects of *Bacillus* sp. P3 on cellulolytic enzymes production, xylan decomposition, lignin removal and enzymatic saccharification of corn stover

To generalize the complete process for carbohydrate degradation of corn stover pretreated by *Bacillus* sp. P3, a mass balance diagram is shown in Fig. 6. After a 20-day pretreatment, the glucan content of corn stover was increased by 51% compared to that of non-pretreated corn stover, while the xylan content was decreased by 35%. As shown in Fig. 6, the lignin content was also decreased, indicating that the strain P3 is able to degrade lignin. Moreover, the solid recovery was 66%, which has met the standard of industrial production (Uppugundla et al. 2014). Our results indicated that the pretreated corn stover containing more accessible glucan could be applicable to the process of fermentation for biofuel production (Guo et al. 2017a). Furthermore, the total amount of reducing sugar released in the process of pretreatment and enzymatic saccharification was 20.58 g from per 100 g of pretreated corn stover, which was 57% higher than those of non-pretreated one. Although this study did not present the integrated technoeconomic evaluations in a process context for the bacterial pretreatment using the strain P3, it provided useful insights to ameliorate the pretreatments in an easier and greener way.

## Conclusions

This study demonstrated an effective strategy to enhance the efficiency of enzymatic hydrolysis using *Bacillus* sp. P3 strain to pretreat the corn stover. After a 20-day pretreatment of corn stover by the strain P3, the reducing sugar yield from the pretreated corn stover after enzymatic saccharification was increased by 56% compared to that from non-pretreated corn stover. Moreover, as a thermoalkalotolerant cellulolytic enzyme-producing bacterium, the strain P3 and its enzymes can not only be utilized to pretreat biomass alone, but can also potentially be combined with other harsher methods to improve the cost-efficiency and eco-friendliness of industrial bioenergy productions.

### Supplementary Information

**Additional file 1: Fig. S1.** Cellulolytic enzyme activities of *Bacillus* sp. P3 cultivated with 0.5% (w/v) corn stover as substrate under different temperature (A) and pH (B) conditions for 36 h. Bars indicate the standard deviation (*n* = 3). **Fig. S2.** Pearson correlation analysis for the final yields of reducing sugar and content of glucan.

## Data Availability

The data and the materials are all available in this article and additional document file.
