# Supplementary material for: Biological pretreatment of corn stover for enhancing enzymatic hydrolysis using Bacillus sp. P3
Source: Bioresour Bioprocess. 2021 Sep 27;8(1):92. doi: 10.1186/s40643-021-00445-8 (PMC8550775; doi:10.1186/s40643-021-00445-8)
Supplement: Supplementary file 1 — Additional file 1: Fig. S1. Cellulolytic enzyme activities of Bacillus sp. P3 cultivated with 0.5% (w/v) corn stover as substrate under different temperature (A) and pH (B) conditions for 36 h. Bars indicate the standard deviation (n = 3). Fig. S2. Pearson correlation analysis for the final yields of reducing sugar and content of glucan. [file 40643_2021_445_MOESM1_ESM.docx]

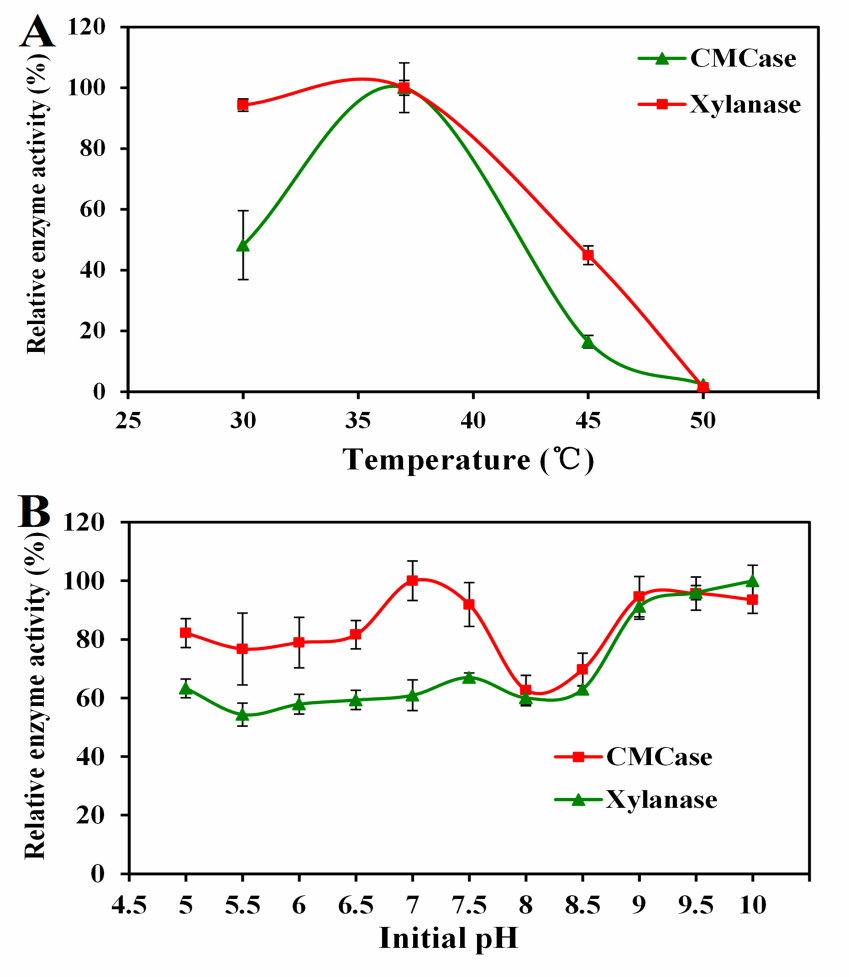


**Fig. S1** Cellulolytic enzyme activities of *Bacillus* sp. P3 cultivated with 0.5% (w/v) corn stover as substrate under different temperature (A) and pH (B) conditions for 36 h. Bars indicate the standard deviation (n = 3).

**Fig. S2** Pearson correlation analysis for the final yields of reducing sugar and content of glucan.
